# Motivational Drivers in Implant Therapy: A Novel Treatment Motivation Scale

**DOI:** 10.7759/cureus.64788

**Published:** 2024-07-18

**Authors:** Krishetha R, Sunanda Rao, Ravishankar PL, Prem Blaisie Rajula, Gayathri K, Kalaivani V, Saravanan A.V., Mohamed Rashik, Sindhujaa R, Sheryl Dolly.A

**Affiliations:** 1 Periodontology, SRM Kattankulathur Dental College and Hospital, SRM Institute of Science and Technology, Chengalpattu, IND

**Keywords:** questionnaire, validation, motivation scale, intrinsic motivation, implant therapy, extrinsic motivation

## Abstract

Aim

This study aims to assess patient motivation during implant therapy using the newly developed implant treatment motivation scale.

Materials and methods

A questionnaire study was conducted, consisting of 15 questions designed to explore the motivating factors behind patients' decision to undergo implant therapy. A total of 50 patients about to undergo implant treatment at the Departments of Periodontology, Oral and Maxillofacial Surgery, and Prosthodontics participated in the study.

Results

Analysis revealed a consistently high level of motivation (intrinsic and extrinsic) among all patients undergoing implant therapy.

Conclusions

The findings highlight the crucial role of motivation in treatment-seeking behavior, emphasizing the importance of dentists in motivating and guiding patients through the process of implant therapy.

## Introduction

Dental implants are regarded as a highly significant advancement in contemporary dentistry. Dental implants have emerged as a preferred option for restoring oral function in patients with missing teeth. The field of dentistry has witnessed significant advancements since the introduction of titanium implants for intraoral use in the late 1970s [1]. For various reasons, including superior comfort, function, appearance, durability, and the preservation of neighboring tooth structures, osseointegrated dental implants are frequently preferred to conventional dentures. Dental implant treatment offers a potentially more acceptable outcome, especially for individuals who are unable to adjust to traditional dentures. With a claimed 90% 10-year survival rate, this therapy approach is comparatively predictable [2].

Patients consider esthetics to be one of the primary benefits of implants over removable and partial dentures [3]. This raises the question of how well-informed the general population is about the advantages of bone-anchored prostheses in terms of functionality [4].

Research has shown that the basic desire for better comfort and ease, reduced physical suffering, and enhanced well-being drives motivation [5]. This drive originates in the physiological, behavioral, cognitive, and social domains. In addition, patients' perceptions and motivation significantly influence their treatment-seeking behavior. Numerous theories and models, like the self-determination theory and the push-pull model, have attempted to explain the different extrinsic and intrinsic factors influencing patients' willingness to accept or reject therapy [6]. The push-pull model, for example, posits that human motivations and behaviors are fundamentally driven by two primary forces: the pursuit of desirable outcomes (pull factors) and the avoidance of undesirable outcomes (push factors) [7].

Patients' values and preferences, the doctors' heuristics and biases, the ambiguities surrounding diagnosis and treatment, and the financial implications all play a role in implant dentistry decisions. The high cost, the participants' distrust in dentists, the intrusive procedures and related complications, and their poor desire to make sizeable additional payments discourage them from contemplating dental implants. Understanding the reasons for apprehensions around dental implants could facilitate efficient communication between healthcare professionals and patients, enabling them to make informed selections. Enhanced communication techniques among dentists can help bridge knowledge gaps, provide patients with understandable and legally tenable information, and help patients set reasonable expectations for the results [4].

In order to establish the abovementioned results, the assessment of motivation using a questionnaire becomes invaluable. We must evaluate the validity and reliability of any new measuring device before implementing it. The objectives of the study were to determine the validity and reliability of the dental treatment motivation scale (DTMS) adjusted to suit implant patients.

## Materials and methods

Ethical clearance, study design, and population

Before data collection, the study was presented to the Institutional Ethical Committee, SRM Institute of Science and Technology. The study commenced after obtaining the ethical clearance certificate. We recorded the monthly inflow at 458 and selected 10% of this population as the sample size for the pilot study. All the participants in the study were given a comprehensive explanation of the study, and informed consent was obtained prior to data collection.

This is a cross-sectional questionnaire study (DTMS scale) conducted among patients attending the outpatient department (OPD) in the Department of Periodontics, Department of Prosthodontics, and Department of Oral and Maxillofacial Surgery over a period of two months from November 2023 to January 2024 [6]. The study population included 50 edentulous and partially edentulous patients aged 18-55 who were interested in undergoing implant rehabilitative therapy and were willing to participate in the study.

The inclusion criteria are as follows: (1) edentulous and partially edentulous patients and (2) patients above 18 years and interested in implant therapy. The exclusion criteria included (1) patients who are not willing to participate.

Study instrument

The data collection instrument included sociodemographic questions as well as the DTMS questionnaire, which was specifically adjusted for implant treatment. The DTMS is a condensed version of the Self-Regulation Questionnaire for dental treatment and comprises 15 Likert scale questions. Seven questions (1, 2, 5, 7, 10, 13, 15) assess intrinsic motivation, while eight questions (3, 4, 6, 8, 9, 11, 12, 14) assess extrinsic motivation.

This scale measures both autonomous and controlled motivations for adopting a healthy attitude toward treatment. Responses are organized on a Likert scale from 1 to 5, ranging from “strongly disagree” to “strongly agree.” Each dimension's score is determined by summing the responses for the items within that dimension and calculating the total score.

Statistical tool and analysis

The obtained data was tabulated and analyzed using Stata software version 18 (StataCorp LLC, College Station, Texas). Descriptive statistics and frequency distributions were analyzed. Cronbach’s alpha, including item deletion, was used for reliability analysis for internal consistency to determine if removing any item would affect reliability. The content validity ratio (CVR) was determined with the aid of thoroughly screened subject matter experts to assess whether the knowledge of each item was essential or not. Repeatability, or test-retest reliability, was evaluated to determine how closely successive measurements of the same variable agreed with each other.

## Results

The study population included 50 patients, 25 males and 25 females, who needed prostheses for missing teeth. The motivation scale’s Cronbach’s alpha of 0.88 indicated that the scale was a reliable tool. All items showed a good degree of total correlation, around 0.9 (Table 1).

**Table 1:** The questionnaire is composed of 15 items in which seven (Q no: 1, 2, 5, 7, 10, 13 & 15) and eight (Q no: 3, 4, 6, 8, 9, 11, 12 & 14) questions assess intrinsic and extrinsic motivation, respectively, and reliability analysis for the questionnaire using the Cronbach's alpha reliability coefficient

Item	Corrected item-total correlation	Alpha
1. I feel that I want to take responsibility for my own health	0.7742	0.9462
2. Others would be furious if I did not do it	0.7910	0.9459
3. I have carefully thought about it and believe it is very important for many aspects of my life	0.7779	0.9462
4. My dentist asked me to do so	0.7892	0.9459
5. I personally believe that it is the best thing for my dental health	0.7473	0.9468
6. I feel pressure from others to do so	0.6814	0.9480
7. I would feel guilty if I didn’t do it	0.7581	0.9465
8. I want others to approve of me	0.7613	0.9465
9. I want the dentist to think I am a good patient	0.5361	0.9508
10. It is easier to do it rather than to think about it	0.6104	0.9494
11. I don’t want others to be disappointed in me	0.4072	0.9531
12.It improves my social acceptability	0.7239	0.9472
13. I would feel bad about myself if I didn’t do it	0.5853	0.9499
14. I want others to see I can do it	0.6467	0.9487
15. It feels good to keep my oral cavity as clean as possible	0.7276	0.9471
Intrinsic motivation	0.9501	0.9426
Extrinsic motivation	0.9557	0.9425
Total motivation	0.9985	0.9416

Cronbach's alpha	Cronbach's alpha based on standardized items	No. of items
0.8836	0.9498	18

CVR measures whether an item is essential. CVR varies between 1 and −1, and a higher score indicates greater agreement among panel members. Options for each item are graded as 1 = essential, 2 = essential, but not useful, and 3 = not useful: CVR = (Ne - N/2)/(N/2), where Ne = number of panelists indicating an item as "essential” and N = total number of panelists. A CVR was generated for each item, and all 15 items had a CVR of 1.00, which indicates a high level of agreement between the members (Table 2).

**Table 2:** Content validation ratio (CVR) Ne: Number of panelists indicating an item as "essential"; CVR: content validation ratio

Question	Expert 1	Expert 2	NE	CVR
1	1	1	2	1
2	1	3	1	1
3	1	1	2	1
4	1	1	2	1
5	1	1	2	1
6	1	1	2	1
7	1	1	2	1
8	1	1	2	1
9	1	1	2	1
10	1	2	2	1
11	1	1	2	1
12	1	1	2	1
13	1	1	2	1
14	1	2	1	1
15	1	1	2	1

Questionnaire data evaluation

The distribution of subjects based on gender and age revealed that 50% were female, with most participants (36%) falling within the 26-35 year age group (mean = 33.26 + 10.67 years) (Table 3). Analysis of the questionnaire responses indicated a high degree of treatment motivation for items 1, 2, 13, 14, and 15, while item 11, "I feel pressure from others to do so," elicited a neutral response. The subjects exhibited high levels of intrinsic, extrinsic, and total motivation (Table 4). A comparison of the mean intrinsic and extrinsic motivation scores showed that there was no statistically significant difference (p-value = 0.08) (Table 5).

**Table 3:** Basic demographic and distribution of subjects based on age and gender

Mean age (years)	33.26 ± 10.67
Sex	Male-25
	Female-25

Sex	15-25 years	26-35 years	36-45 years	46-55 years	Total
Male (%)	32	32	32	4	100
Female (%)	24	40	16	20	100
Total (%)	28	36	24	12	100

**Table 4:** Average values and dispersion of answers to items that evaluate treatment motivation

Total 50 respondents	Mean	SD	Category
1. I feel that I want to take responsibility for my own health	4.32	0.95	Strongly agree
2. Others would be furious if I did not do it	4.28	0.99	Strongly agree
3. I have carefully thought about it and believe it is very important for many aspects of my life	4.18	0.98	Agree
4. My dentist asked me to do so	3.82	1.04	Agree
5. I personally believe that it is the best thing for my dental health	3.94	0.97	Agree
6. I feel pressure from others to do so	3.82	1.06	Agree
7. I would feel guilty if I didn’t do it	4.18	0.84	Agree
8. I want others to approve of me	3.86	0.88	Agree
9. I want the dentist to think I am a good patient	3.94	1.09	Agree
10. It is easier to do it rather than to think about it	4.06	0.95	Agree
11. I don’t want others to be disappointed in me	3.14	1.42	Neutral
12.It improves my social acceptability	4.18	0.77	Agree
13. I would feel bad about myself if I didn’t do it	4.38	0.72	Strongly agree
14. I want others to see I can do it	4.28	0.85	Strongly agree
15. It feels good to keep my oral cavity as clean as possible	4.28	0.75	Strongly agree
Intrinsic motivation	3.90	0.69	Agree
Extrinsic motivation	4.20	0.66	Agree
Total motivation	4.04	0.65	Agree

**Table 5:** Comparison of scores between intrinsic and extrinsic motivation M: Mann-Whitney U test; NS: not significant

Variable	Intrinsic motivation	Extrinsic motivation	p-value
Mean score	31.22	29.44	0.08^M ^(NS)

## Discussion

The current questionnaire is an adaptation of the DTMS [6]. The present study aimed to evaluate the motivational factors influencing patients who require implant therapy for missing teeth. Our findings indicate a balanced representation of genders, with an equal number of male and female participants and a predominant age group of 26-35 years. This demographic detail is crucial as it highlights the typical age range of individuals seeking implant-supported prosthetic solutions.

The reliability of the motivation scale, indicated by a Cronbach’s alpha of 0.88, confirms that the instrument used for measuring motivation was reliable. This is an essential aspect of the study, ensuring that the responses obtained are consistent and reflective of true motivational levels. Moreover, the questionnaire's content validity was strong, with all 15 items achieving a CVR of 1.00. This unanimity among the panel members underscores the relevance and essentiality of the items included in the motivation scale, thereby affirming its content validity. 

Analysis of the questionnaire responses revealed that items 1, 2, 13, 14, and 15 were associated with a high degree of treatment motivation. These items pertain to intrinsic factors, which align with self-determined and autonomous reasons for seeking prosthetic treatment. The results align with a prior study that showed the significant influence of intrinsic motivation on health-related behavior [8]. Conversely, item 11, which stated, "I feel pressure from others to do so," elicited a neutral response. This neutrality indicates that external pressure is not a significant motivational factor for the participants. 

According to the self-determination theory (SDT) [9,10], individuals with an internal perceived locus of causality (PLOC) perceive themselves as the initiators and sustainers of their own actions. In contrast, those with an external PLOC believe that external forces are responsible for initiating, pressuring, or coercing their actions. The study further demonstrated that participants exhibited high levels of both intrinsic and extrinsic motivation, with no significant difference between the two types (p = 0.08). This finding suggests that, while internal desires and self-driven factors play a crucial role, external factors and incentives are equally influential in motivating patients to pursue implant therapy. This balance between intrinsic and extrinsic motivation could be indicative of a comprehensive approach where personal health awareness and rallying from family, friends, or healthcare professionals collectively contribute to the decision-making process [11,12].

 By identifying patients with low motivation early in the treatment process, clinicians can proactively address barriers to adherence and tailor interventions to maximize treatment efficacy and long-term oral health outcomes.

Acknowledging the limitations of our study is crucial, particularly the potential for response bias and the small sample size. Future research should focus on longitudinal studies to explore the dynamic nature of motivation over the course of treatment and investigate the effectiveness of motivational interventions in improving treatment outcomes.

## Conclusions

This study has offered valuable insights into the importance of motivation for patients considering undergoing implant procedures. Through the validation and reliability testing of the Motivation Assessment Scale (MAS), we have demonstrated its utility as a robust instrument for assessing motivation levels in this patient population. By accurately measuring motivation, clinicians can identify patients at risk of poor treatment adherence early in the process, enabling the implementation of targeted interventions to address motivational barriers and enhance treatment efficacy.

Moving forward, it is essential to integrate motivational assessment into routine clinical practice settings. While this study provides valuable insights into the significance of motivation in implant therapy, further research is warranted to explore the dynamic nature of motivation over the treatment continuum and evaluate the effectiveness of motivational interventions in improving long-term outcomes. By continuing to advance our understanding of motivation in periodontal care, we can enhance treatment delivery and ultimately improve the quality of life for patients undergoing implant procedures.
